# Dry beriberi after sleeve gastrectomy: An undiagnosed case report

**DOI:** 10.1016/j.ijscr.2022.107357

**Published:** 2022-06-27

**Authors:** Foolad Eghbali, Mansour Bhahdoust, Hamid Sarafraz, Mobin Naghshbandi, Ali Vaskuyi Eshkevari, Hamidreza Movahedi

**Affiliations:** a Minimally Invasive Surgery Research Center, Division of Minimally Invasive and Bariatric Surgery, Rasool-e Akram Hospital, Iran University of Medical Sciences, Tehran, Iran; b Department of Epidemiology, School of Public Health, Shahid Beheshti University of Medical Sciences, Tehran, Iran; c Student Research Center, Iran University of Medical Sciences, Tehran, Iran

**Keywords:** Beriberi, Gastrectomy, Bariatric surgery, Thiamine Deficiency

## Abstract

**Introduction and importance:**

Sleeve gastrectomy (SG) is a popular surgery for morbid obesity because of minimal complications, while somewhere uncommon micronutrient deficiencies occur that make problems. One of these rare complications is dry beriberi (BB).

**Case presentation:**

A 20-year-old girl with obesity that a few months after SG had non-specific symptoms like nausea, vomiting, debilitating weakness, burning, and tingling in her feet, which led to more tests and imaging and confused the physicians. During the next two months, she had a 40 kg weight loss. The critical test that helped diagnosis was Electromyography and Nerve Conduction Velocity (EMG/NCV), which showed subacute axonal sensory-motor polyneuropathy and decreased level of vitamin B1, proved the patient's diagnosis was dray BB.

**Clinical discussion:**

SG may contribute to vitamin and trace elements deficiency development. One of the micronutrients that deficiencies can disturb the patient is vitamin B1 deficiency. Vitamin B1 deficiencies could be demonstrated with peripheral polyneuropathy, beriberi, or Wernicke-Korsakoff syndrome. Based on decreased vitamin B1 and EMG/NCV results, the diagnosis had been dry beriberi corrected with supplement therapy.

**Conclusion:**

Thiamine deficiency should be suspected in all patients with vomiting, neurological symptoms, and rapid weight loss post-bariatric surgery (BS), even after restrictive surgery. Especially when a patient reports substantial weight loss and vomiting in short order. Hopefully, this case report will make any patient hospitalized with similar conditions evaluated for beriberi and again multivitamin supplementation therapy after SG is emphasized.

## Highlights

• Rapid weight loss after sleeve gastrectomy may cause thiamine deficiency.
• Restrictive surgeries like sleeve gastrectomy may induce thiamine deficiency.
• Multi vitamin supplementation therapy after sleeve gastrectomy is mandatory.

## 1 Introduction

In 2016, >1.9 billion adults worldwide were above a healthy weight (body mass index [BMI] ≥ 25 kg/m^2^), and >650 million of these individuals were living with obesity (BMI ≥ 30 kg/m^2^) [1]. Among weight-loss surgeries, sleeve gastrectomy (SG) surgery is prevalent among people with morbid obesity. During this surgery, a portion of the stomach is removed, resulting in restriction of food consumption and reduced secretion of ghrelin, promoting weight loss [2]. Compared to traditional bariatric surgeries, SG has a significantly lower risk of malnutrition since intestinal bypass is not performed [3]. One of the abovementioned uncommon malnutrition syndromes that may occur after sleeve surgery is the vitamin B1 deficiency, also known as beriberi (BB). BB is a type of peripheral polyneuropathy demonstrated by motor and sensory deficit [4]. BB and thiamine deficiency has been described in Asian literature since the 17th century. It is characterized by peripheral neuropathy and muscle weakness, also called “dry” BB, to differentiate it from “wet” BB, with essentially cardiovascular manifestations [5]. Herein, we report a young woman with morbid obesity suffering from more complicated symptoms diagnosed after a while. The patient provided written informed consent to use the data attributed to this case for publication.

## 2 Case presentation

**Fig. 1:**
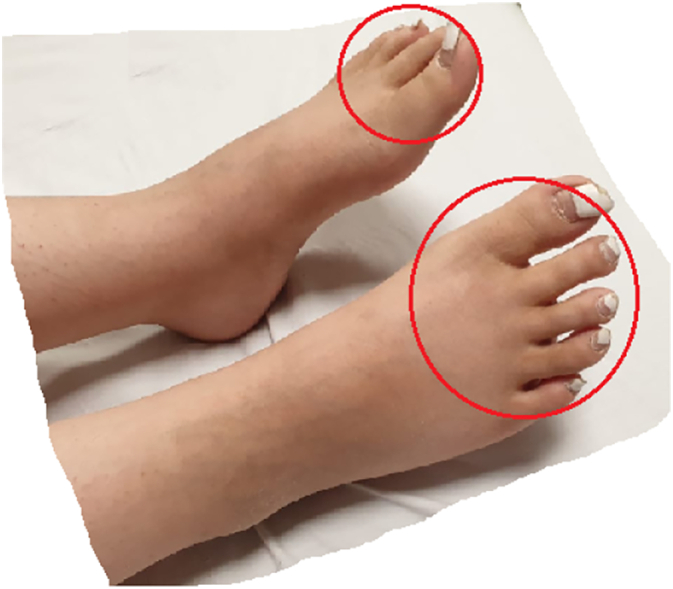
Distal hyperesthesia in legs.

We introduce a 20-year-old female, non-smoker, non-alcoholic with no previous medical disease history recently diagnosed with morbid obesity (weight: 100 kg, height: 168 cm, BMI: 35.43 kg/m^2^). The patient underwent a laparoscopic sleeve gastrectomy in April 2021 by a senior surgeon in private hospital, no early complications were observed, and the patient was discharged after 72 h. During the next two months, the patients had a 40 kg weight loss, resulting in a BMI reduction to 21.26 kg/m^2^. After three weeks, she faced a wave of chronic progressive nausea, vomiting, debilitating weakness, burning, and tingling in her feet. The patient was hospitalized for further medical evaluations. During the first examinations, distal hyperesthesia was found in both legs (Fig. 1). In the lab test, a raised liver function test (LFT) such as (aspartate transaminase (AST): 50 U/L, alanine transaminase (ALT): 80 U/L) was observed, which was relevant with previous proven fatty liver (grade1–2). There was no anemia in complete blood count (CBC). Other lab tests include serum electrolytes (Na, K, P, Ca, albumin, uric acid), erythrocyte sedimentation rate (ESR), C-reactive protein, serum iron, total iron-binding capacity (TIBC), antinuclear Antibody (ANA), anti dsDNA and thyroid function tests were within normal range. Other tests, including viral markers (HCV-Ab, HBs-Ag, HAV-Ab), liver kidney microsome type 1 (anti-LKM-1) antibodies, and anti-smooth muscle antibody (ASMA), were checked to rule out hepatitis, which was normal.

**Fig. 2:**
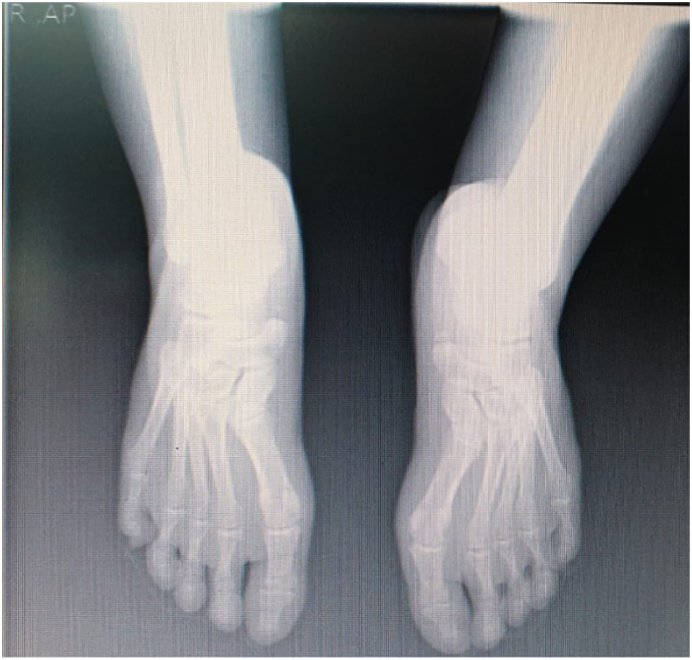
AP/lateral radiography finding.

**Fig. 3:**
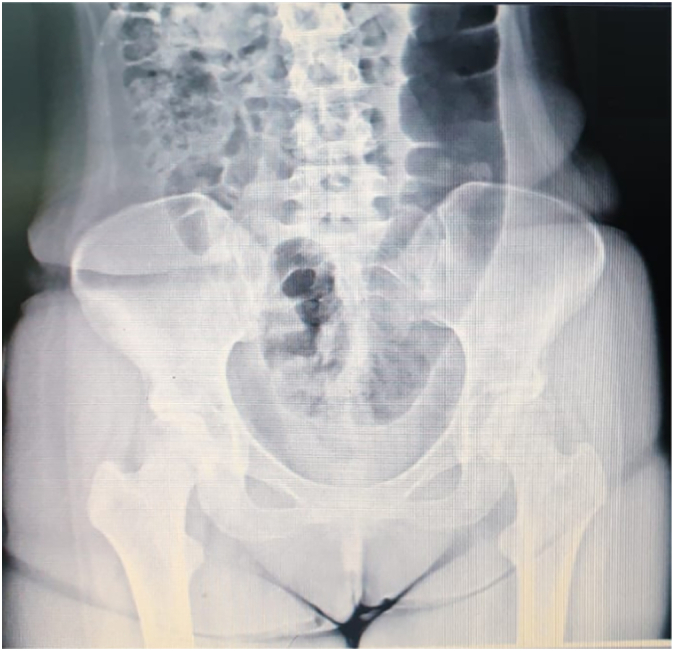
X-ray finding.

Because of severe ankle pain, a normal AP/LAT radiography was performed (Fig. 2). A cervical MRI had done for patients that showed a decreased cervical lordosis that seems not essential and not related to the patient's symptoms. Because of the hyperesthesia, an X-ray and lumbar MRI showed disc bulging in L4-L5, mild central disk protrusion at the L1-L2 level, and decreased disk space (Fig. 3).

Finally, the key test that helped diagnose was EMG/NCV. It showed subacute axonal sensory-motor polyneuropathy. Therefore, the serum level of A, B1, and B12 vitamins, copper, zinc, and ceruloplasmin were checked. The serum level of vitamin B1 was 24 nmol/L which was lower than the normal range (70–180 nmol/L); other tests include zinc serum level (75 μg/dl), vitamin A (0.43 mg/L), ceruloplasmin serum (17.2 mg/dl) and vitamin B12 serum level (>2000 pg/ml) were normal. Based on this decreased level of vitamin B1 and EMG/NCV results, the patient's diagnosis was dry beriberi. We started treating with a high dose of parenteral thiamine (thiamine hydrochloride 200 mg/2 ml daily for ten days) before any carbohydrate because of Wernicke-Korsakoff suspicious, then 100 mg thiamine daily per os for one month [6].

The patient then experienced an improvement as her pain decreased, and she regained sensitivity and strength in her legs. One month after admission, she was discharged after receiving supplements (10 mg/day) and physiotherapy, showing signs of progressive recovery, and her thiamine serum level became normal (70 nmol/L). The work has been reported in line with the SCARE criteria [7].

## 3 Discussion

Gastric resection for malignancy and bariatric surgery for morbid obesity is associated with deficiencies of both macro and micronutrients [8], [9]. Sleeve gastrectomy has been considered to have lower postsurgical vitamin deficiencies prevalence than gastric bypass [10], [11]. SG, however, may also contribute to vitamin and trace deficiency development by allowing food to pass more quickly through the intestines and reducing hydrochloric acid and intrinsic factor production. The effects of SG on nutrient status have been described in several recent studies; however, the results are heterogeneous [12], [13].

In postgastric surgery, possibly due to peripheral neuropathy occurs, the possible causes are vitamin B1, B2, B6, B12, zinc, copper, folate, or vitamin E deficiencies may appear. In some cases, multiple vitamin deficiencies like B group vitamins demonstrate polyneuropathy, numbness, paraesthesia, neuropathic pain, sensory loss, gait disturbance, muscle weakness, or reflex loss [8], [14], [15], [16]. One of the micronutrients that deficiencies can disturb the patient is vitamin B1 deficiency. Reserve this vitamin's contents last nearly 20 days without any additional supplement. The main challenge in patients that underwent sleeve surgery is that approximately 30 % had vitamin B1 deficiency preoperatively [4], [17], [18], while in cases like ours, it is concealed or had not been checked. It is common for obese patients or those undergoing bariatric surgery to be deficient in micronutrients [19], [20]. Typical deficiencies include those of vitamin D, vitamin B6, and calcium [13]. Vitamin B1 deficiencies could demonstrate with peripheral polyneuropathy named beriberi or Wernicke-Korsakoff syndrome. Beriberi usually manifests between 4 and 12 weeks postoperatively with vomiting, but the severity of symptoms is not well understood [21], [22]. However, we need more researches to identify the role of previous chronic gastritis and HP infection in the prevention of an adequate weight loss after SG [23].

Some studies suggested identifying patients at high risk for B1 deficiency preoperatively, including patients with alcohol addiction, patients with an important weight loss in a short period, or patients with protracted vomiting [18], [24].

In our case, maybe previous undiagnosed vitamin B1 deficiency, 40 kg weight loss in a short time, or continuous and chronic vomiting resulted in polyneuropathy due to dry beriberi. Some studies suggested prophylactic parenteral thiamine in all individuals postoperatively who present with dehydration, unable to eat or drink, or chronic vomiting regardless of B1 levels [6], [25].

## 4 Conclusion

It should be suspected in all patients with neurological symptoms in the postoperative period that people who undergo restrictive BS suffer from nutritional deficiencies, including thiamine, especially in cases where a patient reports substantial weight loss and vomiting in short order. Despite the rarity of severe pathology, we should still pay attention to it after BS, take measures to prevent thiamine deficiency, and identify it early. This case report will evaluate any patient hospitalized with similar conditions for beriberi, and again, multivitamin supplementation therapy after sleeve gastrectomy is emphasized.

## Consent

Written informed consent was obtained from the patient for publication of this case report and accompanying images. A copy of the written consent is available for review by the Editor-in-Chief of this journal on request.

## Provenance and peer review

Not commissioned, externally peer-reviewed.

## Ethical approval

This study was approved by the ethics committee of Iran University of Medical Science.

## Funding

N/A.

## Guarantor

Dr. Foolad Eghbali.

## Research registration number

N/A.

## CRediT authorship contribution statement

Foolad Eghbali: Analysis and interpretation of data, drafting the article, final approval of the version to be submitted.
Mansour Bhahdoust: Analysis and interpretation of data, drafting the article, final approval of the version to be submitted.
Hamid sarafraz: Analysis and interpretation of data, drafting the article, final approval of the version to be submitted.
Mobin Naghshbandi: Analysis and interpretation of data, drafting the article, final approval of the version to be submitted.
Ali Vaskuyi Eshkevari: Analysis and interpretation of data, drafting the article, final approval of the version to be submitted.
Hamidreza Movahedi: Analysis and interpretation of data, drafting the article, final approval.

## Declaration of competing interest

The authors declare that they have no competing interests.
